# Partial reduced Pi transport function of PiT-2 might not be sufficient to induce brain calcification of idiopathic basal ganglia calcification

**DOI:** 10.1038/s41598-019-53401-0

**Published:** 2019-11-21

**Authors:** Kazuya Nishii, Ritsuko Shimogawa, Hisaka Kurita, Masatoshi Inden, Michio Kobayashi, Itaru Toyoshima, Yoshiharu Taguchi, Akihiro Ueda, Hidetaka Tamune, Isao Hozumi

**Affiliations:** 1 Laboratory of Medical Therapeutics and Molecular Therapeutics, Gifu Pharmaceutical University, Gifu, Japan; 2 Department of Neurology, National Hospital Organization Akita National Hospital, Akita, Japan; 3 Department of Neurology, Toyama University Hospital, Toyama, Japan; 4 Department of Neurology, Fujita Health University, Aichi, Japan; 5 Department of Neuropsychiatry, Tokyo Metropolitan Tama Medical Center, Tokyo, Japan

**Keywords:** Neurodegeneration, Psychosis

## Abstract

Idiopathic basal ganglia calcification (IBGC) is a rare intractable disease characterized by abnormal mineral deposits, including mostly calcium in the basal ganglia, thalamus, and cerebellum. *SLC20A2* is encoding the phosphate transporter PiT-2 and was identified in 2012 as the causative gene of familial IBGC. In this study, we investigated functionally two novel *SLC20A2* variants (c.680C > T, c.1487G > A) and two *SLC20A2* variants (c.82G > A, c.358G > C) previously reported from patients with IBGC. We evaluated the function of variant PiT-2 using stable cell lines. While inorganic phosphate (Pi) transport activity was abolished in the cells with c.82G > A, c.358G > C, and c.1487G > A variants, activity was maintained at 27.8% of the reference level in cells with the c.680C > T variant. Surprisingly, the c.680C > T variant had been discovered by chance in healthy members of an IBGC family, suggesting that partial preservation of Pi transport activity may avoid the onset of IBGC. In addition, we confirmed that PiT-2 variants could be translocated into the cell membrane to the same extent as PiT-2 wild type. In conclusion, we investigated the PiT-2 dysfunction of four *SLC20A2* variants and suggested that a partial reduced Pi transport function of PiT-2 might not be sufficient to induce brain calcification of IBGC.

## Introduction

Idiopathic basal ganglia calcification (IBGC), also known as Fahr’s disease or prevailingly as primary familial brain calcification, is a rare neurodegenerative disease. Historically, too many nomenclatures have been discussed and used for this condition^1–3^. IBGC is characterized by abnormal mineral deposits, mostly calcium, in brain regions, including the basal ganglia, thalamus, cerebellum, and others. We have investigated the patients in Japan^4,5^. We have 231 patients with 33 familial case now in Japan. *SLC20A2* (IBGC1 was previously named IBGC3) encoding the phosphate transporter PiT-2 was first identified in 2012 as the first causative gene of IBGC^6^. Thereafter, other causative genes of IBGC were continuously discovered, such as *PDGFRB* (IBGC4), *PDGFB* (IBGC5), and *XPR1* (IBGC6)^7–9^. Most recently, *MYORG* is identified as a new causative gene of IBGC and is associated with recessive IBGC^10^. Among these, IBGC1 is the most frequent and accounts for about 30%–50% of familial cases^2,4,5^. Currently, no effective therapy is available for patients with IBGC^2^.

PiT-2 encoded by *SLC20A2* is a sodium-dependent phosphate type III transporter belonging to the inorganic phosphate transporter (PiT) family^11^. This membrane protein consists of 12 transmembrane domains and transports inorganic phosphate (Pi), especially in the brain^12–14^. Several groups, including our group, have reported novel variants in *SLC20A2*^4,6,15–20^. Before the discovery of *SLC20A2* variants in patients with IBGC, Bottger, P. and Pedersen, L. had shown that the artificial replacements E55K, E575K, D28N, and D506N were leading to PiT-2 variants unable to transport Pi into the plasma of *Xenopus laevis* oocytes^21,22^.

Several studies on *SLC20A2* variants have suggested that Pi homeostasis is disrupted as a result of Pi transport dysfunction of PiT-2 causing IBGC^4,6,15,23^. Wang *et al*. showed that the six variants, S601W, S601L, T595M, E575K, G498R, and V42del, of *SLC20A2* in patients with IBGC caused a Pi transport dysfunction of PiT-2 using *Xenopus laevis* oocytes^6^. Furthermore, Yamada *et al*. showed that the function of PiT-2 was impaired by the A51V, R71H, T115M, and S637R variants using PolyPhen-2^4^. Recently, Sekine *et al*. demonstrated a remarkable decreased activity in ^32^Pi intracellular transport using induced pluripotent stem cells (iPSCs) from patients with the *SLC20A2* variant encoding W616X^23^. Many *SLC20A2* variants have been reported so far, but no hotspot of variant sites has been specifically identified^4,6,19,24^.

The ProDom regions I_11_-L_161_ (N-PD1131) and V_492_-V_640_ (C-PD1131) located at the N-terminus and C-terminus of PiT-2, respectively, had been identified as potentially important for maintaining Pi transport activities^25^. Based on this finding, Bottger, P. and Pedersen, L. have shown the importance of PD1131 for maintaining Pi transport activities with artificial replacement or deletion in PiT-2^21,22,26^.

PiT-2 also functions as a receptor for amphotropic murine leukemia virus (A-MuLV), but no correlation between viral receptor activity and phosphate transporter activity has been reported^26–29^.

In the study, we report two novel variants in *SLC20A2* and established stable PiT-2-expressing cell lines with the two novel variants and with two variants reported previously^17,18,30^ to analyze their function in PiT-2 and explore the pathophysiological mechanisms of IBGC. Furthermore, we discuss the possibility of the partial decrease in Pi uptake might not be sufficient for the onset of IBGC.

## Results

### Variants and clinical manifestation

#### Clinical manifestations

The clinical manifestations are summarized in Table 1. A positive family history of IBGC was present in 3 families.

**Table 1:** Clinical features of the nine individuals with *SLC20A2* variants

	Case 1				Case 2	Case 3		Case 4	Case 5
Mutation	c.680C > T	c.680C > T	c.680C > T	−	c.82G > A	c.358G > C	c.358G > C	c.82G > A	c.1487G > A
	A227V	A227V	A227V	−	D28N	G120R	G120R	D28N	C496Y
Zygosity	Hetero	Hetero	Hetero	−	Hetero	Hetero	Hetero	Hetero	Hetero
Exon	6	6	6	−	2	3	3	2	8
**Information**									
Age at detection of calcification, y	−	−	−	64	81	63	46	83	34
Age at onset, y	−	−	−	62	NE	62	43	81	30
Onset symptom	−	−	−	parkinsonism	incidental	gait disturbance	gait disturbance	neurogenic syncope	psychosis
**Neurologic findings**									
Cognitive impairment (MMSE)	−	−	−	normal	25	21	26	28	28
Pyramidal sign	−	−	−	+	+	+	−	−	−
Extrapyramidal sign	−	−	−	+	+	−	−	−	−
**Familiy information (except the proband)**									
No. of other individuals with calcification	−	−	−	1	1	1	1	NE	NE
No. of other individuals with confirmed mutation	−	−	−	0	NE	1	1	NE	NE
No. of other symptomatic individuals	−	−	−	1	1	1	1	−	−
Other symptoms in the familiy	−	−	−	dementia, ataxia	pshyzophrenia	−	−	−	−

Abbreviations: NE = not examined, MMSE = mini-mental state examination.

#### Familial cases. *Case 1 (in family 1)*

**Figure 1:**
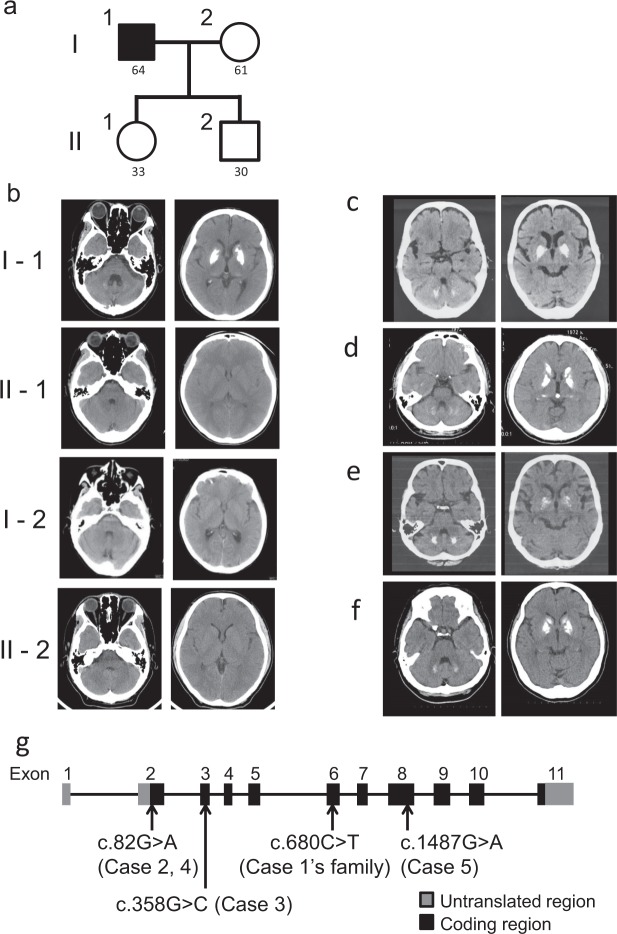
Family tree including Case 1 and CT images of Case 1 proband and his family members, CT images of Case 2, Case 3, Case 4 and Case 5, and schematic representation of *SLC20A2* variants in this study. (**a**) Family tree of Case 1. The ages at which CT scan was taken are shown under symbols. (**b**) CT images of Case 1 proband and his spouse, daughter, and son. (**c**) CT images of Case 2. (**d**) CT images of Case 3 (CT images of a son of the proband). (**e**) CT images of Case 4. (**f**) CT images of Case 5. (**g**) Four variants in exon 2 (c.82G > A, p.D28N), exon 3 (c.358G > C, p.G120R), exon 6 (c.680C > T, p.A227V), and exon 8 (c.1487G > A, p.C496Y) were examined for their functional effects in this study.

The proband (Fig. 1a; I-1) was a 64-year-old man with parkinsonism. Resting tremor in his leg was recognized at the age of 63. He showed hyperreflexia in his extremities and mild rigidity in his lower extremities. However, he apparently did not present cognitive impairment and signs of cerebellar ataxia. His CT images revealed severe calcification at the bilateral basal ganglia and faint calcification at the cerebellum dentate nucleus (total calcification score = 18) (Fig. 1b; I-1). The autopsy report of his elder brother also revealed similar calcification at the age of 79. This autopsy report and family-genetic studies will be published in detail elsewhere. Genetic studies were performed in this family, following the obtainment of written informed consent. Surprisingly, the novel *SLC20A2* variant (c.680C > T, p.A227V) in exon 6 was accidentally found in his wife, son, and daughter, but not in the proband himself (Supplementary Fig. 1a). His wife, son, and daughter had no calcification in the brain (Fig. 1b; I-2, II-1, and II-2) and showed no abnormalities in the neurological examination.

#### Familial cases. *Case 2 (in family 2)*

The patient was an 81-year-old woman and had been referred to the department of neurology for evaluation because of colon cancer. She had suffered from right frontal subcortical hemorrhage 3 years before. She presented with left superficial sensory impairment after the stroke and postural hand tremors. Her CT images revealed severe calcification at the bilateral basal ganglia, mild calcification at the cerebellum dentate nucleus, and faint calcification at the pineal bodies, the choroid plexus, and subcortical white matter (Fig. 1c). Her DNA study identified the variant (c.82G > A, p.D28N) in exon 2 (Supplementary Fig. 1b). This variant was previously reported^17,18^. According to her medical records, her only daughter suffered from schizophrenia and showed calcification at the bilateral globus pallidus (not shown). Her DNA analysis has not been performed yet.

#### Familial cases. *Case 3 (in family 3)*

The proband and her variant in *SLC20A2* have been reported in our previous study^30^. Briefly, this 63-year-old woman presented with bilateral drop feet and gait disturbance due to peripheral neuropathy. The neurological examination showed mild cognitive impairment (MMSE 21), cerebellar ataxia, and spasticity in lower extremities. Her CT images revealed severe calcification at the bilateral basal ganglia and subcortical area, and faint calcification at the thalamus, but none at the cerebellum dentate nucleus (total calcification score = 20, #3 in Fig. 2 of ref.^30^). Her DNA study showed a variant (c.358G > C, p.G120R) in exon 3. Her son had similar clinical manifestations and CT images (Fig. 1d) and carried the same variant in exon 3.

**Figure 2:**
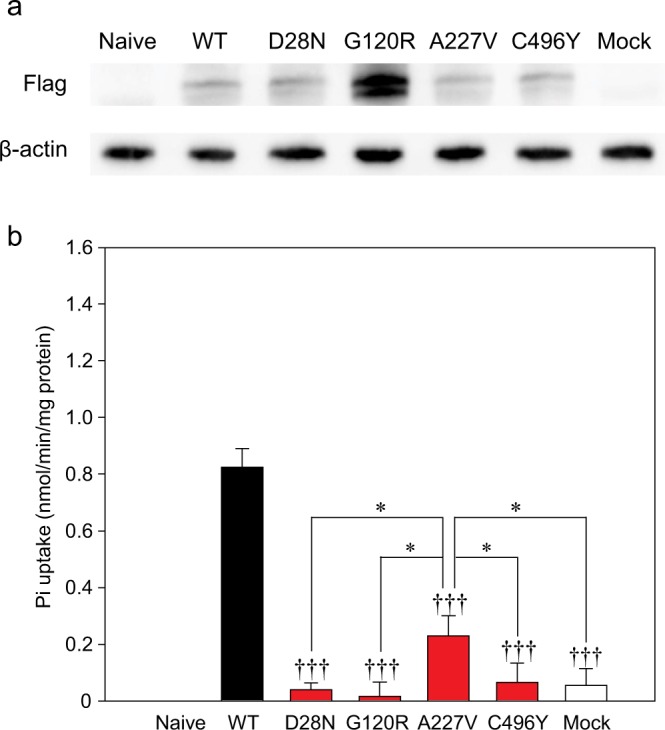
PiT-2 expression and Pi transport activities in PiT-2 missense variants. (**a**) Western blots of PiT-2 expression in Flp-In CHO cells. Full-length blots are presented in Supplementary Fig. 2. (**b**) The Pi uptake assay was performed in Flp-In CHO cells expressing PiT-2 WT or variants. Data were normalized to the Pi uptake in CHO Naive cell. Each value represents the mean ± SEM of n° = ° 8/group relative to mock. The significance of any difference was determined using ANOVA, followed by the Bonferroni/Dunn *post hoc* test (*p < 0.05 versus A227V; ^†††^p < 0.001 versus WT).

#### Sporadic cases. *Case 4*

The patient was an 83-year-old woman who suffered from neurogenic syncope but showed no other neurological abnormalities, such as dementia and extrapyramidal and cerebellar signs. Her CT images revealed moderate calcification at the bilateral basal ganglia and the cerebellum dentate nucleus and faint calcification at the hippocampus (Fig. 1e). Her DNA study revealed the same variant as found in Case 2 (Supplementary Fig. 1b). No family member presented with neurological symptoms or calcification in the brain as far as we did investigate. Case 2 and Case 4 live in the Toyama Prefecture in Japan. However, no blood relationship was found between their families in the detailed family survey.

#### Sporadic cases. *Case 5*

The patient had been reported in a case report written in Japanese^31^. Briefly, this 43-year-old woman suffered from repetitive psychosis, including delusions, hallucinations, disorganized speech, and grossly disorganized behavior. Her symptoms were always relieved with a small amount of risperidone (1.5–2.0 mg/day) within 1 week. Her CT images revealed severe calcification at the bilateral basal ganglia and moderate calcification at the cerebellum dentate nucleus (total calcification score = 24, Fig. 1f). Her DNA study revealed a variant (c.1487G > A, p.C496Y) in exon 8 (Supplementary Fig. 1c). There were no other family members presenting with similar neurological symptoms or calcification in the brain as far as we surveyed.

### Establishment and confirmation of PiT-2 expression in Flp-In Chinese hamster ovary (CHO) cells

Among the four *SLC20A2* variants described in the present study (Fig. 1g), all can influence PiT-2 function based on the PolyPhen-2 software analysis (data not shown). We constructed Chinese hamster ovary (CHO) cells stably expressing PiT-2-WT and PiT-2 variants D28N, G120R, A227V, and C496Y using the Flp-In system. Protein expression was confirmed by western blots (Fig. 2a). We confirmed that proteins were expressed at the same level in all Flp-In CHO cells. However, an extra band appeared under the PiT-2 band in the PiT-2-G120R group that we assumed to be a protein degradation product due to the unstable variant.

### Phosphoric acid transport activities were significantly maintained in the PiT-2-A227V variant that did not show IBGC pathology

We measured Pi transport activities of Flp-In CHO cells using the radioactive tracer ^32^P-H_3_PO_4_ to evaluate whether PiT-2 missense variants had an effect on Pi transport activities. The Pi transport activities were decreased to the mock level in PiT-2-D28N, G120R, and C496Y cells in comparison with that in WT cells (Fig. 2b). However, in PiT-2-A227V cells, the Pi transporter activity significantly increased compared with mock or other variant-expressing cells. In addition, PiT-2-A227V variant showed no IBGC pathology, such as mineralization. Therefore, the Pi transport activity of PiT-2 is a contributing factor causing IBGC pathology.

### PiT-2 missense variants translocated to the membrane evaluated by PiT-2 virus receptor activity

**Figure 3:**
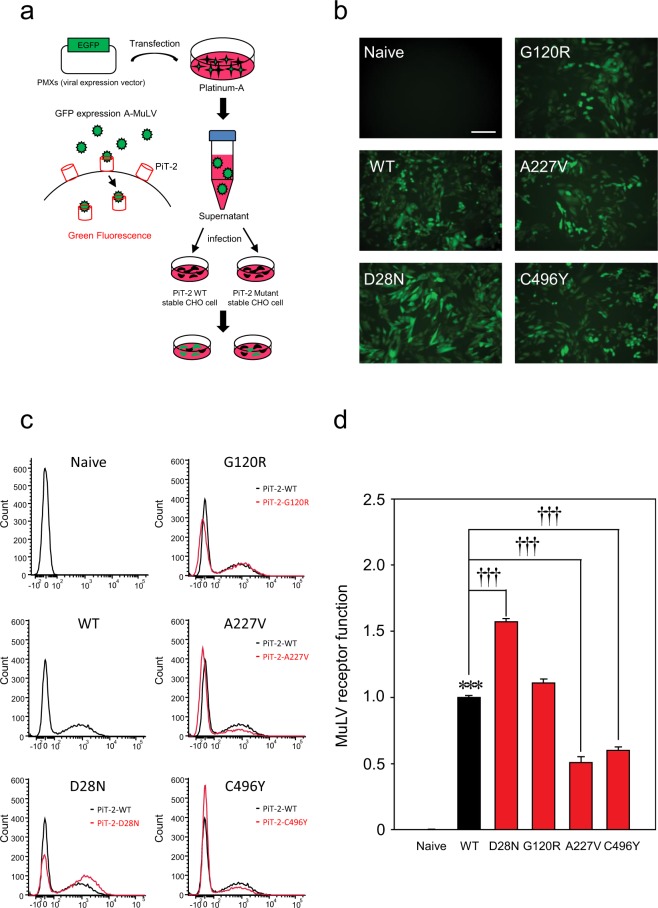
PiT-2 missense variants translocated to the membrane and assessed for A-MuLV receptor function. (**a**) Schematic of A-MuLV receptor function experiment. (**b**) Infected Flp-In CHO cells were photographed by all-in-one microscope at 72 h. Scale bar, 20 µm in A. (**c,d**) A-MuLV receptor functions were assessed by FACS analysis. Black = PiT-2-WT; red = PiT-2 variants. Data are relative to PiT-2-WT representing 1. Each value represents the mean ± SEM of three determinations, relative to Naive. The significance of any difference was determined using ANOVA, followed by the Bonferroni/Dunn *post hoc* test (***p < 0.001 versus Naive; ^†††^p < 0.001 versus WT).

In a previous report, PiT-2 virus receptor activity was measured to evaluate protein folding and membrane translocation of PiT-2^26^. Here, we evaluate the effects of PiT-2 variants on PiT-2 folding and membrane translocation using A-MuLV receptor functions. The green fluorescence protein (GFP) gene was introduced into the viral vector to co-express GFP protein in Flp-In CHO cells infected with A-MuLV (Fig. 3a). The number of GFP fluorescent cells was counted by FACS analysis in 10,000 cells, and the viral receptor function was calculated as the ratio of GFP-positive cells to 10,000 cells. Cells were photographed using a fluorescence microscope before FACS analysis (Fig. 3b). PiT-2 viral receptor functions were significantly higher in PiT-2-D28N and did not change in PiT-2-G120R cells as compared with WT, respectively (Fig. 3c,d). On the other hand, viral receptor functions were reduced by half in PiT-2-A227V and C496Y cells (Fig. 3c,d).

### PiT-2 membrane translocation was also shown in PiT-2 missense variants evaluated by HiBiT System

**Figure 4:**
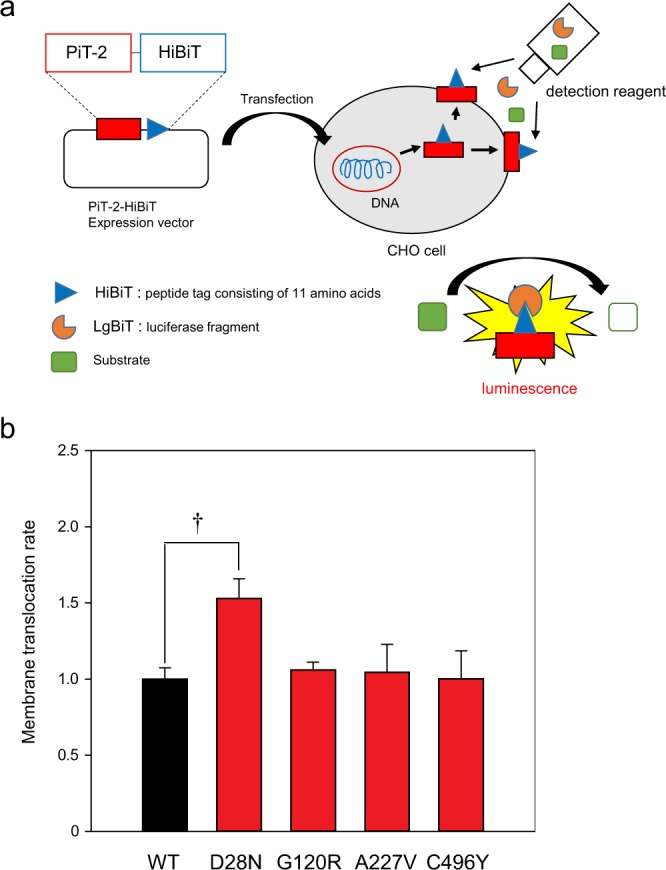
PiT-2 membrane translocation was present in PiT-2 missense variants by HiBiT Extracellular System. (**a**) Schematic of the HiBiT Extracellular System. (**b**) Measurement of the membrane translocation rate using the HiBiT Extracellular System. Data were normalized to the mock-transfected cells and corrected to PiT-2-WT representing 1. Each value represents the mean ± SEM of three determinations, relative to the Naive. The significance of any difference was determined using ANOVA, followed by the Bonferroni/Dunn *post hoc* test (^†^p < 0.05 versus WT).

We evaluated the membrane migration of PiT-2 variants more deeply using the HiBiT System in accordance with a previous report^23^. Membrane translocation rates of PiT-2 were measured in cells transiently expressing HiBiT-tagged PiT-2-WT or variants to investigate the effect of PiT-2 missense variants on membrane translocation. HiBiT is a small tag protein consisting of 11 amino acids that binds to LgBiT and emits luminescence by the substrate. Since LgBiT is cell membrane impermeable, protein can be quantified on the membrane surface (Fig. 4a). Membrane translocation rate was calculated as the ratio of membrane protein expression level to total protein expression level. The membrane translocation rate was significantly higher in PiT-2-D28N cells than PiT-2-WT cells (Fig. 4b). In contrast, there was no significant difference of membrane translocation rate in other PiT-2 variant-expressing cells in comparison with PiT-2-WT cells (Fig. 4b).

### PiT-2 topology model and variant distributions

**Figure 5:**
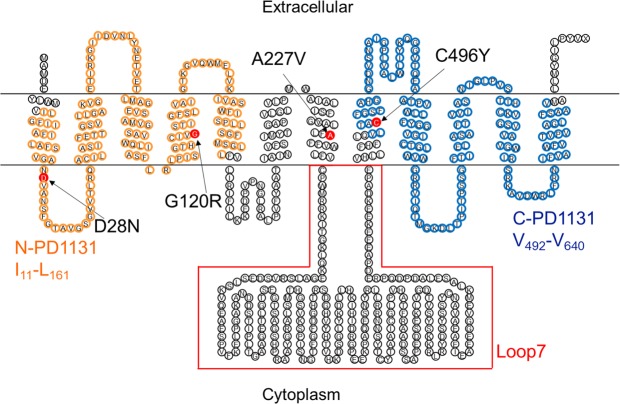
Topology model of PiT-2 protein created with TOPO2. The I_11_-L_161_ (N-PD1131) region and the V_492_-V_640_ (C-PD1131) region are highlighted in orange and blue, respectively. PiT-2 variants reported in this paper are highlighted in red.

A PiT-2 topology model was created using the transmembrane protein display software (TOPO2). The region of I_11_-L_161_ (N-PD1131) and the region of V_492_-V_640_ (C-PD1131) were emphasized with orange and blue, respectively. Variants reported here are shown in red. Whereas PiT-2-D28N, G120R, and C496Y variants were found in the PD1131 region, the PiT-2-A227V variant was not. (Fig. 5).

## Discussion

We have previously reported six new variants in *SLC20A2* (four missense variants, one nonsense variants, and one frameshift variants). The four missense variants were predicted to have deleterious damages of protein function in the *in silico* analysis using PolyPhen-2^4^. In addition, missense variants showed impairment in the Pi transport activity in CHO cells stably expressing them and in endothelial cells developed from patient’s iPSCs^23^.

In this study, we have reported two new missense variants in *SLC20A2* (c.680C > T, p.A227V, and c.1487G > A, p.C496Y) with their clinical manifestation. One new missense variant was found by chance in the clinically non-affected persons of the family 1 genetic study. Four variants (including the variant c.680C > T, p.A227V) were predicted to cause deleteriousness of protein function based on the PolyPhen-2 result. We performed a functional study using Flp-In CHO cells expressing PiT-2 variants.

Interestingly, the Pi transport activity in PiT-2-A227V cells was maintained at 27.8% of the level of PiT-2-WT cells, whereas in D28N, G120R, and C496Y variants, the activities were lower than 10% of the control level. These results indicate that PiT-2 dysfunction caused by variants leads to the pathogenesis of IBGC and suggest that a partial decrease in Pi uptake might not be sufficient for the onset of IBGC.

PiT-2 is a 12-pass transmembrane protein and sodium-dependent phosphate transporter of type III^11^. A single nucleotide variant of *SLC20A2* from IBGC patients can cause loss of Pi transport activity^4,6,15^. The PD1131 region in the N-terminus and the C-terminus of PiT-2 was reported as essential for Pi transport activity^25,26^, and each single variant of *SLC20A2* reported previously^6^ was found in the PD1131 region. Among the variants studied, PiT-2-D28N and G120R are located in the N-PD1131 region on the N-terminal side, and PiT-2-C496Y is located in the C-PD1131 region on the C-terminal side. Three variants of this study influenced the Pi transport activity and manifested typical clinical features resulting from phosphorus homeostasis impairment. On the other hand, the single nucleotide variant A227V, found in the IBGC patient’s families presenting no IBGC clinical features, showed 27.8% of the normal Pi transport activity.

A previous report had shown that deletion of the L183-R254 region of PiT-2, encompassing the A227V variant position, showed a diminished the Pi transport activity^26^. The differences between A227V variant and L183-R254 deletion^26^ are likely derived from the different mechanisms of their transporter activities.

Interestingly, the retention of the Pi transport activity in the A227V variant was correlated with no clinical manifestations and no findings in brain CT images, suggesting that a partial decrease in Pi uptake might not be sufficient for the onset of IBGC.

In this study, although the *SLC20A2* variants found in IBGC diminished the Pi transport activity, the patients carried heterozygous variants, and both wild-type PiT-2 and variant PiT-2 are theoretically expressed in the body. The mechanism by which *SLC20A2* variants lead to IBGC has not yet been elucidated. *SLC20A2* variants could lead to either haploinsufficiency or dominant negative effects. Based on the haploinsufficiency hypothesis, half of *SLC20A2* expression would decrease the Pi transport activity^32^. On the other hand, a study suggested the dominant negative effect of the *SLC20A2* variants encoding D28N, H502A, and E575K on the Pi transport activity^33^. The effect of *SLC20A2* variants in IBGC remains, however, controversial. The higher levels of Pi detected in CSF of patients with IBGC, especially *SLC20A2* variants, suggest the disease progresses with the dyshomeostasis of Pi transport in the brain.

The originally reported PiT-2 function was as a retrovirus receptor on the cell surface^26–29^. However, the correlation between viral receptor activity and Pi transport remains unclear. We assessed the gamma-retroviral receptor function and found that although viral receptor functions were altered by variants of PiT-2, the activity varied between 50% and 160% of WT levels in all variants. Furthermore, the translocation of PiT-2 to the cell surface was assessed using the HiBiT technology to measure the membrane transfer rate of WT PiT-2 and PiT-2 variants. The membrane translocation rate of PiT-2-D28N was significantly increased, whereas those of G120R, A227V, and C496Y were similar to WT. Although the virus receptor function mediated by D28N seems related to membrane surface relocation, the relationships among virus receptor function, Pi transport activity, and/or clinical symptoms remain to be elucidated.

In addition, PiT-2 was reported to have additional functions, including sensing extracellular Pi concentrations and modulating neurite outgrowth^34,35^. However, the relationship between these functions and IBGC pathology has not been recognized. The neurite outgrowth is thought to be associated with loop 7, which is a large intracellular domain of PiT-2^34^. In addition, Kimura *et al*. have shown that in patients carrying the S637R variant of PiT-2, the expression level of PiT-2 is clearly low in the regions of high PiT-2 expression, including the frontal cortex, putamen, and cerebellum^36^. Taglia *et al*. have shown that in PiT-2 trp626_629dup patients, PiT-2 does not migrate to the membrane and is abnormally localized to the nucleus^37^. Yamada *et al*. have suggested that clinical features in patients with IBGC are similar according to the variant sites. In Case 3, both the proband and her son showed neuropathy. Whereas the diverse effects of PIT-2 variants should be clarified, other factors, such as environment and disease-modifying genes, should be considered.

In conclusion, we have reported two novel pathogenic variants in PiT-2 and showed their associated dysfunction using CHO cells. Interestingly, in the A227V variant, the Pi transport activity partially remained, and no clinical manifestations of IBGC were found in the carriers. On the other hand, this suggests that the partial increase of PiT-2 activity may lead to improved manifestation in patients with IBGC.

## Materials and Methods

### Subjects and samples

We collected clinical information on patients with IBGC in a nationwide study supported by a grant for research on intractable diseases from the Ministry of Health, Labor and Welfare of Japan. The diagnostic criteria were described previously^4^. Briefly, patients with causative biochemical abnormalities, including calcium, inorganic phosphate, and intact parathyroid hormone (iPTH), were excluded. The genetic survey and all experiments on human blood samples were approved by the Ethics Committees of Gifu University and Gifu Pharmaceutical University and performed in accordance with Ethical Guidelines for Medical and Health Research Involving Human Subjects in Japan, and Ethical Guidelines for Human Genome/Gene Analysis Research in Japan. After written informed consent was obtained, peripheral blood samples were collected. This study was registered to the UMIN Clinical Trials Registry approved by International Committee of Medical Journal Editors (UMIN000030100).

### Sequence analysis

Genomic DNA was extracted from the patient’s blood samples using DNA Quick II (Genomic DNA Isolation kit; DS Pharma Biomedical Co., Ltd.). PCR amplification was performed using the primers whose sequences are listed in Supplementary Table 1. Primers were designed from the NCBI Probe database. For PCR, 0.4 µL template DNA, 0.4 µL of 10 µM forward and reverse primers, 1.6 µL dNTP, 2.0 µL of 10 × Ex Taq buffer, and 0.1 µL Ex Taq HS were mixed with 15.1 µL sterile water. The thermal cycling conditions were as follows: preamplification denaturation (1 cycle), 94 °C for 30 s; amplification (total of 30 cycles), 98 °C for 10 s, Table 1 listed temperature for 30 s, 72 °C for 30 s; and final elongation (1 cycle), 72 °C for 2 min. PCR products were purified with illustra ExoStar (GE Healthcare Life Sciences). The purified PCR products were used for sequencing with an ABI BigDye Terminator v3.1 Cycle Sequencing Kit (Applied Biosystems) using M13 forward or reverse primers. After ethanol precipitation, automated sequencing was performed on an ABI 3100 Genetic analyzer using both the forward and reverse primers. Variants found in dbSNP, the 1000 Genomes Project database, or the Exome Variant Server database of the National Heart, Lung, and Blood Institute Exome Sequencing Project were excluded.

### Cell culture

The CHO and Flp-In CHO cell lines were cultured in Ham’s F-12 medium supplemented with 10% (v/v) fetal bovine serum according to the manufacturer’s protocol (Thermo Fisher Scientific). The Platinum-A (Plat-A) cell line was cultured using Dulbecco’s Modified Eagle Medium supplemented with 10% (v/v) FBS.

### DNA plasmid vector

Human *SLC20A2* cDNA (NM_006749) was purchased from Thermo Fisher Scientific and subcloned into a p3 × FLAG-CMV-14 vector (Sigma-Aldrich) with the flag tag inserted at C-terminal between the HindIII/EcoRI sites (PiT-2-flag vector). The *SLC20A2* with the flag tag was subcloned into the pcDNA5/FRT vector between the HindIII/NotI sites (Thermo Fisher Scientific, pcDNA5/FRT-PiT-2-flag vector). Subsequently, the *SLC20A2* variant (D28N, G120R, A227V, C496Y) was generated using the PrimeSTAR® Mutagenesis Basal Kit (Takara Bio Inc.) according to the manufacturer’s protocol. For HiBiT analysis, the *SLC20A2* variant (D28N, G120R, A227V, C496Y) and wild-type *SLC20A2* were transferred into the pBiT3.1-C vector (Promega) between the HindIII/EcoRI sites using restriction enzymes. The primer set sequences are listed in Supplementary Table 2. The A-MULV expression vector was constructed from pMXs and pEGFP-N1 vectors (Clontech). EGFP was subcloned into pMXs vector between the BamHI/NotI sites (pMXs-EGFP).

### Flp-In system

Flp-In CHO cells were seeded in six-well plates and co-transfected with pOG44 and pcDNA5/FRT-PiT-2-flag (WT, D28N, G120R, A227V, C496Y) or GFP (mock) according to the manufacturer’s protocol (Thermo Fisher Scientific). Hygromycin B-resistant cells were selected and cloned for experiments.

### Western blotting

Cells were washed with PBS and lysed in 200 µL RIPA buffer (20 mM Tris-HCl, pH = 7.5, 150 mM NaCl, 1 mM EDTA, 1% NP-40, 0.1% sodium deoxycholate, 0.1% SDS, 10 µg/mL aprotinin, 10 µg/mL leupeptin, and 1 mM phenylmethylsulfonyl fluoride). Cell extracts were incubated on ice for 20 min and then centrifuged at 14,000 × g for 30 min at 4 °C. Supernatants were collected as protein samples, mixed with SDS sample buffer and incubated overnight at 4 °C. Protein concentration was determined using the Pierce BCA protein Assay Kit (Thermo Fisher Scientific). Proteins were separated by 10% SDS-PAGE for 50 min and transferred to PVDF membrane for 90 min. Blocking was performed with Blocking One (Nacalai Tesque Inc.) for 1 h at room temperature (RT). After blocking, the membrane was incubated at 4 °C overnight with the following mouse monoclonal primary antibodies: ANTI-FLAG M2 (1:1000, Sigma) and β-actin (1:1000, Santa Cruz Biotechnology) dissolved in 5% Blocking One/TBS-T (50 mM Tris-HCl, pH 7.6, 150 mM NaCl, 0.5% Tween-20), and 5% skim milk, respectively. After incubation, the membrane was incubated with the goat anti-mouse secondary antibody conjugated with HRP (1:2000, Santa Cruz Biotechnology) dissolved in 3% Blocking One/TBS-T or 3% skim milk for 60 min at RT. The membrane was incubated in ECL Prime (GE Healthcare) to generate chemiluminescence from HRP antibodies, and chemiluminescence was detected using LAS3000 Mini (Fujifilm). Band density was measured using ImageJ software.

### Phosphate uptake assay

All phosphate uptake assays were performed essentially as described^38^. The transport rate was expressed as nmol Pi per minute per mg protein.

### Virus infection

Plat-A cells were seeded in 10 cm dishes and transfected with pMXs-EGFP. Cell culture supernatant was collected and added to the Flp-In CHO cells. Cells were lysed in PBS. The amphotropic receptor function was analyzed by measuring GFP expression cell count rates using a BD FACSVerse flow cytometer (BD Biosciences).

### HiBiT extracellular system

CHO cells were seeded in 96-well plates and transiently transfected with pBiT3.1-C-PiT-2 (WT, D28N, G120R, A227V, C496Y, or mock). Translocation rates of PiT-2 were measured using the Nano-Glo HiBiT Extracellular Detection System according to the manufacturer’s protocol (Promega). Briefly, HiBiT is a small tag protein of 11 amino acids that binds to LgBiT, and the HiBiT–LgBiT complex emits luminescence upon substrate binding. Because LgBiT is impermeable to the cell membrane, only the HiBiT–LgBiT complex on the cell membrane surface is detected. The membrane translocation rate was calculated as the ratio of the membrane protein expression level to the total protein expression level. The transfection efficiency of each vector was confirmed *via* the co-transfection of GFP. Nano-Glo HiBiT Extracellular Reagent was prepared in advance and added to the transfected CHO cells. Cells were incubated for 10 min, and luminescence of the membrane protein was measured using the GloMax Multi Detection System (Promega). After measurement, cells were lysed with Triton-X100, and total protein was measured.

### Statistical evaluation

Data are given as the mean ± standard error of the mean (SEM). The significance of differences was determined by an analysis of variance (ANOVA). Further statistical analysis for *post hoc* comparisons was performed using the Bonferroni/Dunn test (Stat View).

Supplementary Information accompanies this paper at doi:

## Supplementary information

Supplemental information
