# Supplementary material for: Partial reduced Pi transport function of PiT-2 might not be sufficient to induce brain calcification of idiopathic basal ganglia calcification
Source: Sci Rep. 2019 Nov 21;9:17288. doi: 10.1038/s41598-019-53401-0 (PMC6872723; doi:10.1038/s41598-019-53401-0)
Supplement: Supplementary file 1 — Supplemental information [file 41598_2019_53401_MOESM1_ESM.pdf]

Supplemental Information

Partial reduced Pi transport function of Pit2 might not be sufficient to induce brain calcification of idiopathic basal ganglia calcification

Kazuya Nishii[1], Ritsuko Shimogawa[1], Hisaka Kurita[1], Masatoshi Inden[1], Michio Kobayashi[2], Itaru Toyoshima[2], Yoshiharu Taguchi[3], Akihiro Ueda[4], Hidetaka Tamune[5] & Isao Hozumi[1]

[1] Laboratory of Medical Therapeutics and Molecular Therapeutics, Gifu Pharmaceutical University, Gifu, Japan

[2] Department of Neurology, National Hospital Organization Akita National Hospital, Akita, Japan

[3] Department of Neurology, Toyama University Hospital, Toyama, Japan

[4] Department of Neurology, Fujita Health University, Aichi, Japan

[5] Department of Neuropsychiatry, Tokyo Metropolitan Tama Medical Center, Tokyo, Japan

Correspondence and requests for materials should be addressed to I. H. (email: hozumi@gifu-pu.ac.jp, Tel & Fax: +81-58-230-8121)

Supplementary Table 1 Primer sequences for variants analysis of *SLC20A2*

Primer	Temperature (°C)	Sequence (5' to 3')
Exon-2-F	50	CATGCCAAAGTTTAGATCCCA
Exon-2-R	50	AGAAAATAAATGGTTGCCTGA
Exon-3-F	62	CGCTTTGTAAAGAAACAATTCACA
Exon-3-R	62	GCTCACGCCCTCTAATCCTG
Exon-4-F	55	GTCAGCTCTGCCAAGTCA
Exon-4-R	55	ACAATTATTCCTCTAACCCCTC
Exon-5-F	55	CAACAGTGGGCTCTTTGACA
Exon-5-R	55	TTACTATCAGCCAACAACTCC
Exon-6-F	52	TTTAAGCACATATTCGCCAGA
Exon-6-R	52	GCACTCAGGAACGGAGAGAC
Exon-7-F	60	CACCTGGCCTCAACTTCATT
Exon-7-R	60	TGAGCCTGCTCTGCTGACTA
Exon-8-F	60	GGCATGGTGTCGCGCTTGTAG
Exon-8-R	60	CCGGCGACCTCCTAGTTGT
Exon-9-F	60	CCGCGGCTGTAGTCTCAATTA
Exon-9-R	60	GGGGCCTGTTTAAGTCTGTGC
Exon-10-F	60	GCGGCCTCTTGTTCTGTTAAAAT
Exon-10-R	60	CCCGGAGACCTGGAGAACCT
Exon-11-F	58	GCTGAAGAGAAGAATGGGGAAAG
Exon-11-R	58	GGTGAACAGTGTGGGATGGAG

Supplementary Table 2 Primer pairs used for polymerase chain reaction.

Primer	Sequence (5' to 3')
PiT-2-flag (Forward)	GTTAAGCTTATGGCCATGGATGAGTATTTG
PiT-2-flag (Reverse)	GCAGAATTCGCCACATATGGAAGGATCCCATA
pcDNA5/FRT-PiT-2-flag (Forward)	GTTAAGCTTATGGCCATGGATGAGTATTTG
pcDNA5/FRT-PiT-2-flag (Reverse)	GCAGCGGCCGCGGATGCCACCCGGGATCACTA
D28N (Forward)	TGCAAACAATGTTGCCAACTCCTTTG
D28N (Reverse)	GCAACATTGTTTGCACCAACAGAAAA
G120R (Forward)	CATTGTGCGTTCTACTATAGGATTCT
G120R (Reverse)	GTAGAACGCACAATGCAGTGCGTTCC
A227V (Forward)	CTGTTCGTTTTTTTGTGTGGCTCTT
A227V (Reverse)	AAAAAAAACGAACAGGAGGGCGACAC
C496Y (Forward)	ACCGCCTATTTCGGGTCCTTTGCTCA
C496Y (Reverse)	CCCGAAATAGGCGGTGAGGACCTGCA

Supplementary Fig.1

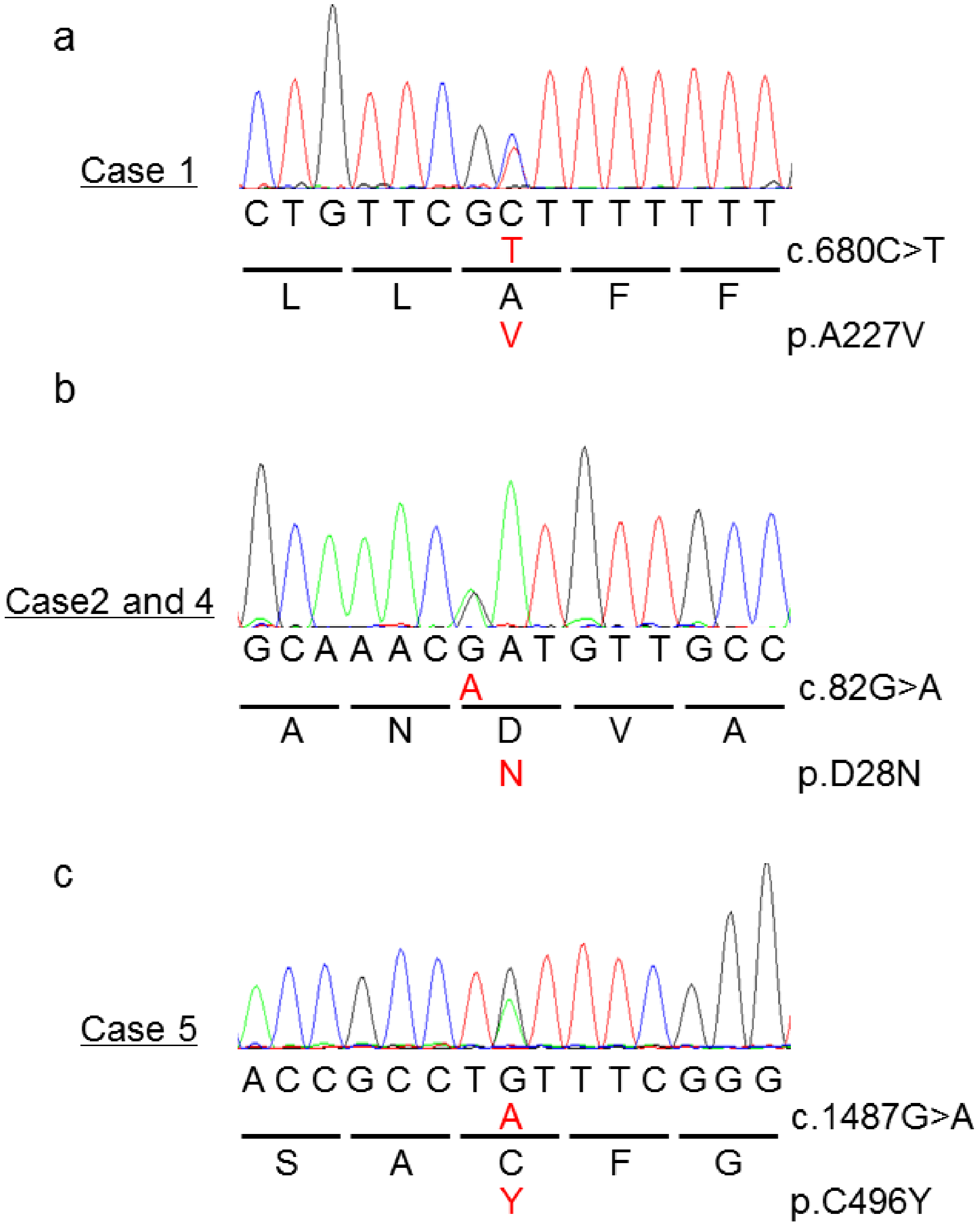

Supplementary Fig.2

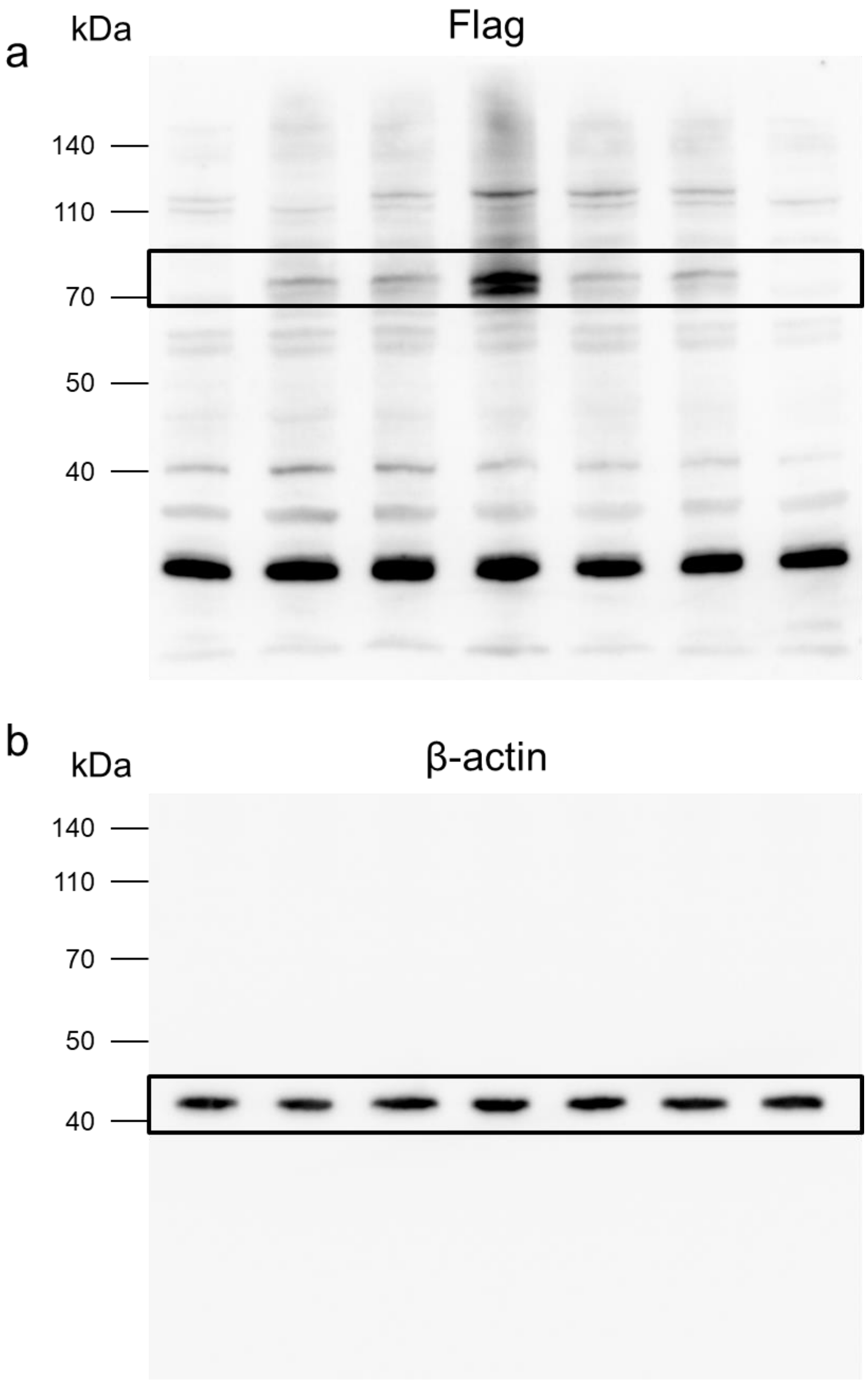

Figures and legends

Supplementary Figure 1. DNA sequence electropherograms showing different heterozygous mutations in *SLC20A2* identified in IBGC patients. Letters in red indicate mutation sequences.

(a) A variant (c. 680C>T) of *SLC20A2* found in Case 1's families. (b) A variant (c.82G>A) of *SLC20A2* found in Case 2 and Case 4. (c) A variant (c.1487G>A) of *SLC20A2* found in Case 5.

Supplementary Figure 2. Full length blots of Fig 2a.

(a) Full-length blots of Anti-Flag (Fig. 2a) is presented above. (b) Full-length blots of Anti-β-actin (Fig. 2a) is presented above. The area of cropped blot presented in Fig. 2a is displayed with a black square.
